# Link-Polymorphism of 5-HTT Promoter Region Is Associated with Autoantibodies in Patients with Systemic Lupus Erythematosus

**DOI:** 10.1155/2016/3042726

**Published:** 2016-10-13

**Authors:** Shu Li, Shuang Liu, Fan Chen, Yuqi Cheng, Ru Bai, Aiyun Lai, Zhaoping Lu, Jian Xu

**Affiliations:** ^1^Department of Rheumatology and Immunology, First Affiliated Hospital of Kunming Medical University, Kunming, Yunnan 650032, China; ^2^Department of Internal Medicine, Yunnan Red Cross Gaoxin Hospital, Kunming, Yunnan 650032, China; ^3^Department of Psychiatry, First Affiliated Hospital of Kunming Medical University, Kunming, Yunnan 650032, China

## Abstract

Serotonin transporter linked polymorphic region (5-HTTLPR) was reported to associate with depression in systemic lupus erythematosus (SLE) patients by our team. To explore whether 5-HTTLPR plays a role in the pathogenesis of SLE, we tested 138 SLE patients and 138 age and sex matched health controls (HCs) for 5-HTTLPR by polymerase chain reaction (PCR) and agarose gel electrophoresis. Interestingly, the results suggest that the frequencies of SS genotype and S allele in SLE patients with positive anti-Sm antibody and anti-U1RNP antibody were both significantly higher than the other genotypes and alleles. However, the frequencies of 5-HTTLPR genotypes and alleles were of no significant difference between SLE patients and HCs. This suggested that 5-HTTLPR was not a high-risk susceptible gene in SLE but might relate to SLE by affecting production of some autoantibodies, especially anti-Sm and anti-U1RNP antibody.

## 1. Introduction

Systemic lupus erythematosus (SLE) is an autoimmune disease mediated by multiple genetic and environmental factors. Complicated interactions of genetic and molecular factors are involved in the pathogenesis of SLE. SLE is featured by a variety of autoantibodies produced by B cells derived from abnormal activation and differentiation. B cells can attract and present autoantigens to T cells through special immunoglobulins on their surfaces. Abnormalities like dysfunction of T and B cells, proliferation of B cells, and dominance of T helper (Th) 2 cells are involved in the pathogenesis of SLE. Meanwhile, multiple cytokines such as tumor necrosis factor alpha (TNF-*α*), interleukin-10 (IL-10), IL-4, and chemokines appear and cause inflammation in tissues [1].

Serotonin (5-HT) plays an important regulatory role in all kinds of immune responses mediated by T cells, B cells, and macrophages through direct and indirect ways. It participates in autoimmune reaction by mediating the activation of T and B cells in several pathways [2]. Serotonin transporter (5-HTT or SERT) locates on the presynaptic membrane and regulates the reuptake of 5-HT in synaptic cleft. The serotonin transporter linked polymorphic region (5-HTTLPR) modifies 5-HTT function in both transcription and translation levels and affects the ability of taking in and releasing 5-HT procedures of 5-HTT; thus, it regulates the concentration of 5-HT eventually. 5-HT combines the corresponding serotonin receptor (5-HTR) and affects the activity of 5-HTT in return. This study was designed to find out the relationship between serotonin system and SLE.

## 2. Material and Methods

### 2.1. Subjects

138 SLE patients were recruited from April 2011 to May 2012 from inpatient and outpatient centers of the Department of Rheumatology and Immunology of First Affiliated Hospital of Kunming Medical University, Kunming, Yunnan, China, a member unit of Chinese SLE Treatment and Research Group (CSTAR). They were all diagnosed with SLE according to the 1997 revised American College of Rheumatology (ACR) criteria for the classification of lupus [3].

The inclusion criteria included the following factors: (1) diagnosis of SLE according to the 1997 revised ACR criteria for the classification of lupus; (2) capability of reading and writing; (3) being between the ages of 15 and 60; (4) voluntary writing consent of this study from patients or statutory guardians.

The exclusion criteria included the following factors: (1) patients with diagnosis of rheumatoid arthritis (RA), systemic sclerosis (SSc), Sjogren's syndrome (SS) (primary or secondary), other connective tissue diseases (CTD), or drug induced SLE; (2) patients with severe disorders of major organs such as heart, liver, or kidney; (3) patients with present or previous diagnosis of neurological or psychiatric disease; (4) patients with kidney failure or other pathologic conditions which may induce encephalatrophy (e.g., stroke, hypertension, diabetes mellitus, and addiction to alcohol or drugs); (5) patients with present or previous diagnosis of epilepsy; febrile convulsion in childhood is not included.

138 health controls (HCs) were recruited in the Physical Examination Center from the same hospital. All HC group members received thorough physical examinations to exclude SLE and other major diseases excluded in the SLE group.

Prior to entry into the study, each participant provided written informed consent after receiving a complete description of the study. All the participants were Chinese Han population. This research was approved by the Institutional Review Board of Kunming Medical University, Yunnan Province, China (ClinicalTrials.gov: NCT00703742).

### 2.2. Collection of Population Statistics and Clinical Data

Demographic and clinical data of both SLE patients and HCs were collected, including name, sex, age, marriage status, education background, profession, family history, and medical history. Disease status like symptoms, physical signs, and durations of disease was also recorded in SLE patients.

### 2.3. Test of Autoantibodies

Anti-nuclear antibody (ANA) was tested by indirect immunofluorescence assay (IFA) method with ANA (Hep-2) IFA kit (Xinsaier Technology Co., Ltd., Kunming, China). ANA spectrum was detected by line immunoassay (LIA) method with ANA spectrum linear immunoassay kit (IMTEC Company, Berlin, German).

### 2.4. Test of 5-HTTLPR

(1) DNA extraction: extract DNA from 5 mL peripheral venous blood anticoagulated by 2% EDTA with the TaKaRa MiniBEST Universal Genomic DNA Extraction Kit Ver.4.0 D824A (TaKaRa Biotechnology Co., Ltd., Dalian, China); (2) polymerase chain reaction (PCR) amplification of target genes: sense primer (5′- ATG CCA GCA CCT AAC CCC TAA TGT -3′) and antisense primer (5′- GG ACC GCA AGG TGG GCG GGA -3′) were synthesized by Sangon Biotech Co., Ltd., Shanghai, China. In a 50 *μ*L nuclease-free microcentrifuge tube, the following were combined according to the manufacturer's instructions (TaKaRa LA Taq DRR002A, TaKaRa Biotechnology Co., Ltd., Dalian, China): template DNA (2.5 ng), dNTP mixture (8 *μ*L), PCR Buffer II (Mg^2+^ Plus) (5 *μ*L), sense primer (1 *μ*L), antisense primer (1 *μ*L), and sterilized saline in a 50 *μ*L reaction volume. Touchdown PCR procedures were carried out in the following manner: initial denaturation at 95°C for 5 minutes, denaturation at 95°C for 30 seconds, annealing at 62°C for 30 seconds, and extension at 72°C for 1 minute; 26 cycles were performed; the temperature was then held at 72°C for 10 minutes. The PCR products were compared with DL2000 DNA Marker as reference. (3) Genotype reading: the PCR products were separated by 2% agarose gel electrophoresis (voltage 180 V, 20 min) and were observed under ultraviolet (UV) gel imaging system Gel DocEQ (Bio-Rad Laboratories, Inc., CA, USA). Imaging analysis detected gene segments of 419 bp and 375 bp. The 375 bp was designated as the “S” or “short” variant, while the 419 bp was designated as the “L” or “long” variant. Thus, the three possible genotypes were “SS” homozygote, “LS” heterozygote, and “LL” homozygote.

### 2.5. Statistical Analysis

Statistical analysis was conducted with SPSS 17.0 (SPSS Inc., 1989–2004). Descriptive statistics and chi-square test were used to describe and compare the differences of genotype and allele frequency between SLE patients and HCs and within the SLE patients. Odds ratio (OR) and confidence interval (CI) were calculated and the results were statistically significant when *p* < 0.05. All statistical tests were two-sided.

## 3. Results

### 3.1. Demographical and Clinical Data

This research involved 138 SLE patients in which 18 were males and 120 were females; they were from 16 to 52 years old, on average 30.51 ± 8.58 years old; their education varied from 5 to 19 years, on average 11.65 ± 3.47 years. 138 HCs were matched by age and sex, and their education varied from 6 to 20 years, on average 15.13 ± 2.75 years. Their education level was comparable (*p* > 0.05).

### 3.2. Autoantibody Levels in SLE Patients

Several autoantibodies were detected in SLE patients. All patients were ANA positive. There were 75 patients (54.3%) with positive anti-double stranded (ds) DNA antibody; 80 patients (57.9%) with positive anti-Sm antibody; 68 patients (49.2%) with positive anti-nucleosome antibody; 41 patients (29.0%) with positive anti-U1 ribonucleoprotein (RNP) antibody; 79 patients (57.2%) with positive anti-histone antibody; 59 patients (42.7%) with positive anti-ribosomal P0 antibody; 62 patients (44.9%) with positive anti-SSA 52 kD antibody; 89 patients (64.5%) with positive anti-SSA 60 kD antibody; 39 patients (28.2%) with positive anti-SSB antibody.

### 3.3. Hardy-Weinberg Genetic Equilibrium Test

The 5-HTTLPR genotype frequency of both the SLE patients and HCs was consistent with the Hardy-Weinberg Equilibrium.

### 3.4. 5-HTTLPR Genotypes in SLE Patients and HCs

**Figure 1:**
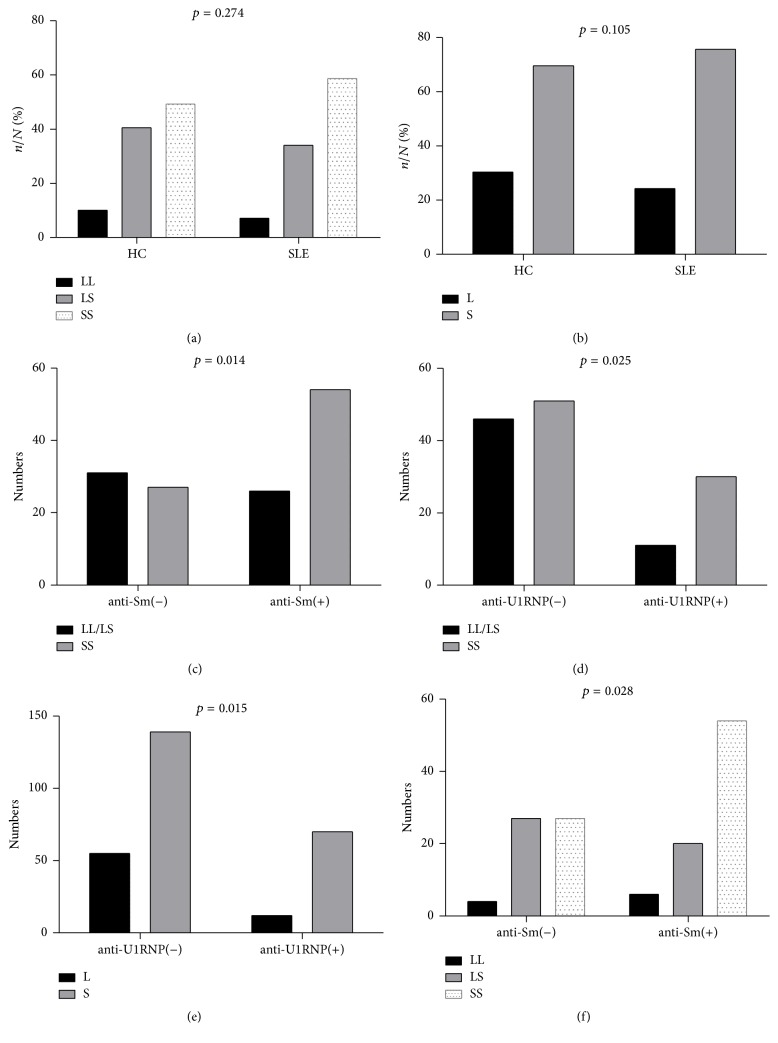
5-HTTLPR genotypes in SLE patients and HCs. (a) The frequency of 5-HTTLPR genotypes in HCs and SLE patients; (b) the frequency of 5-HTTLPR alleles in HCs and SLE patients. (c) The frequency of SS genotype was significantly higher than LL/LS genotype in SLE patients with positive anti-Sm antibody; (d) the frequency of SS genotype was significantly higher than LL/LS genotype in SLE patients with positive anti-U1RNP antibody; (e) the frequency of S allele was significantly higher in SLE patients with positive anti-U1RNP antibody; (f) the frequency of SS genotype was significantly higher than the other 2 genotypes in SLE patients with positive anti-Sm antibody.

The frequency of SS genotype was 58.7% in SLE patients and 49.3% in HCs, while the frequency of LL was 7.2% in SLE patients and 10.1% in HCs; thus, the frequency of LS was 34.1% and 40.6% in each group (*χ* ^2^ = 2.587, *p* = 0.274) (see Figure 1(a)). The frequency of S allele was 75.7% and 69.6% in SLE patients and HCs, respectively, while the L allele was 24.3% and 30.4% in corresponding groups (*χ* ^2^ = 2.635, *p* = 0.105) (see Figure 1(b)). Both genotypes and alleles were not significantly different between the SLE patients and HCs (*p* > 0.05).

In SLE patients with positive anti-Sm antibody, the frequency of SS genotype was significantly higher than LL/LS genotype (*p* = 0.014, OR = 2.385, 95% CI 1.188–4.785) (see Figure 1(c)); the same result was also found in SLE patients with positive anti-U1RNP antibody (*p* = 0.025, OR = 2.46, 95% CI 1.108–5.461) (see Figure 1(d)). In patients with all the other antibodies, no significant differences were found.

In SLE patients with positive anti-U1RNP antibody, the frequency of S allele was significantly higher than L allele (*p* = 0.015, OR = 2.308, 95% CI 1.161–4.591) (see Figure 1(e)). In patients with all the other antibodies, no significant differences were found.

In SLE patients with positive anti-Sm antibody, the frequency of SS genotype was significantly higher than the other 2 genotypes (*p* = 0.028) (see Figure 1(f)). In patients with all the other antibodies, no significant differences were found.

## 4. Discussion

5-HT is usually considered as a typical neurotransmitter. It also plays a regulatory role in the immune system. 95% of 5-HT in peripheral circulation are synthesized by pheochromocytes in the gastrointestinal tract and then released to blood. 5-HTTs of platelets take in and store 5-HT in platelet dense granules, which composes the main pool of 5-HT. There are several studies which revealed that 5-HTTLPR could regulate the expression of 5-HTT and affect the 5-HT of synaptic cleft afterwards. 5-HTTLPR is the main regulatory region of 5-HTT expression and modifies its function at transcription and translation levels [4]. The S allele-dominant variant downregulates the expression of 5-HTT and leads to low levels of uptake and release of 5-HT, while the L allele-dominant variant acts the opposite way. The transcriptional activity of L allele-dominant variant is 3 times that of S allele [5]. S allele limits the transcriptional activity of 5-HTTLPR and leads to low expression of 5-HTT. Lesch and Merschdorf found that the transport activity of 5-HT of L allele was higher than S allele [6]. 5-HT can induce the upregulation of 5-HT1A receptor and proliferation of T and B cells by activating mitogen. 5-HT is also associated with increase of translocation of nuclear factor-kappa B (NF-*κ*B) in cell nucleus [7]. Müller et al. found that 5-HT could increase the production of proinflammatory factors, such as IL-6, IL-4, and IL-10, and decrease the levels of Th1 cell-related cytokines and IL-12p70 in dendrite cells (DCs). Meanwhile, 5-HT upregulates the secretion of Th2 chemoattractant CCL22 and downregulates the Th1 chemoattractant CXCL10 in DCs by binding to 5-HTR4 and 5-HTR7. DCs induce Th2 dominance in CD4^+^ T cell through 5-HT [8]. Lood et al. found that SLE patients had decreased 5-HT levels in serum and platelets compared with HCs. This decrease was related to severe disease subtypes such as lupus nephritis (LN), and it might be mediated by type I interferon [9]. Our team has found the significant hypomethylation of promoter region of 5-HTR1A (PR-HTR1A) and higher expression of 5-HTR1A mRNA in SLE patients [10]. Birmingham et al. found that self-perceived stress of patients with LN carrying the 5-HTR1A-1019 G allele could increase the risk of lupus flares, although 5-HTTLPR was not associated with lupus flares [11]. These studies suggested that the regulation of 5-HT system might involve the pathogenesis of SLE.

There are several studies about 5-HTTLPR and other autoimmune diseases. One study indicated that depressed women with SS genotype had higher levels of thyroid stimulating hormone (TSH) and worse antidepressant response compared to LL/LS genotypes [12]. A Japanese clinical trial showed that, in women with diabetes mellitus, hypercholesterolemia, and hypertension, SS genotype showed a larger increase in blood glucose after meals than fasting blood glucose (FBG) [13]. A recent study showed that, in patients with type 2 diabetes mellitus (T2DM), the frequencies of SS and LS genotypes were higher than HCs. S allele might play a role in the pathogenesis of T2DM [14]. An RA animal study showed that, with no intervention, monkeys with S allele had significantly higher neutrophil-to-lymphocyte ratios. 5-HTTLPR might be a unique marker of autoimmune inflammation [15]. Ferreira et al. found that although 5-HTTLPR was not associated with osteoporosis, another gene polymorphism serotonin transporter with a variable number of tandem repeats (5-HTTVNTR) was associated with osteoporosis [16]. Sikander et al. found that the LL genotype of 5-HTTLPR was associated with microscopic colitis and ulcerative colitis and serotonin levels were significantly higher in those patients compared to HCs [17]. Colucci et al. found no association of 5-HTTLPR with irritable bowel syndrome (IBS), while the LS and SS genotypes were significantly correlated with IBS symptom severity [18]. Yamakawa et al. found that S allele of the 5-HTTLPR was associated with stress reactivity like increased heart rate, secretion of IL-1*β*, and cortisol levels in multilevel stress-related biological systems [19]. The studies above indicated that 5-HTTLPR was involved in the pathogenesis of several autoimmune diseases. There is no research about 5-HTTLPR and the pathogenesis of SLE yet.

Our study showed that the frequencies of 5-HTTLPR genotypes and alleles were of no significant difference in SLE patients and HCs of our center, which might suggest that 5-HTTLPR is not a high-risk susceptible gene of SLE and more researches are needed.

During the progression of SLE, B lymphocytes proliferate and produce large amounts of autoantibodies which target different parts of cells such as nucleus, cell membrane, and cell plasma. These autoantibodies, including ANA, anti-dsDNA, anti-Sm, and anti-U1RNP, not only are the consequence of disorders of immune system but also are aggravating the disease. In 1979, Lerner and Steitz confirmed that protein Sm and RNP were both located at small nuclear ribonucleoprotein (snRNP) [20]. In snRNP, Sm is located at U1, U2, U4, and U5 snRNP. The most commonly used anti-RNP antibody usually only binds to U1 part, so we call it anti-U1RNP antibody. Since they share the same antigen epitopes, anti-U1RNP antibodies are sometimes concurrent with anti-Sm antibodies [21]. In SLE patients, anti-Sm antibody is highly specific and associated with disease activity and certain subtypes such as LN and neuropsychiatric SLE (NPSLE). Some researchers believed that anti-Sm antibody was more related to nephropathy than anti-dsDNA antibody [22–24]. Anti-U1RNP antibody can be positive in several autoimmune diseases, but it is considered to be a symbolic autoantibody in mixed connective tissue disease (MCTD) [25]. In MCTD patients, 95% are anti-U1RNP antibody positive in high titers with anti-Sm antibody negative. In other autoimmune diseases such as SLE, only 20–40% patients are anti-U1RNP antibody positive with anti-Sm antibody positive. It is still controversial whether anti-U1RNP antibody is associated with SLE disease activity. Asian and African populations have higher prevalence of anti-U1RNP antibody. Some studies showed that SLE patients with NPSLE, LN, pulmonary fibrosis, or pericarditis were usually with positive anti-Sm/U1RNP antibodies. Anti-U1RNP antibody is also believed to relate to Raynaud's phenomenon and dermal involvement [26–31].

This study showed that the frequencies of SS genotype and S allele in SLE patients with positive anti-Sm and anti-U1RNP antibody were significantly higher. The specific mechanism is not clear and it may suggest that SS homozygote and S allele are associated with the production of anti-Sm and anti-U1RNP antibody. More related studies, including tests of serotonin and B cell numbers and functions, are warranted.

Based on the researches above, we conclude that 5-HTTLPR might not be a high-risk susceptible gene in SLE. However, it may relate to SLE by affecting the autoantibodies generation, especially anti-Sm antibody and anti-U1RNP antibody.
